# Structural basis of RNA recognition by the SARS-CoV-2 nucleocapsid phosphoprotein

**DOI:** 10.1371/journal.ppat.1009100

**Published:** 2020-12-02

**Authors:** Dhurvas Chandrasekaran Dinesh, Dominika Chalupska, Jan Silhan, Eliska Koutna, Radim Nencka, Vaclav Veverka, Evzen Boura

**Affiliations:** 1 Institute of Organic Chemistry and Biochemistry of the Czech Academy of Sciences, Prague, Czech Republic; 2 Department of Cell Biology, Faculty of Science, Charles University, Prague, Czech Republic; Washington University School of Medicine, UNITED STATES

## Abstract

Severe acute respiratory syndrome coronavirus 2 (SARS-CoV-2) is the causative agent of the coronavirus disease 2019 (COVID-19). SARS-CoV-2 is a single-stranded positive-sense RNA virus. Like other coronaviruses, SARS-CoV-2 has an unusually large genome that encodes four structural proteins and sixteen nonstructural proteins. The structural nucleocapsid phosphoprotein N is essential for linking the viral genome to the viral membrane. Both N-terminal RNA binding (N-NTD) and C-terminal dimerization domains are involved in capturing the RNA genome and, the intrinsically disordered region between these domains anchors the ribonucleoprotein complex to the viral membrane. Here, we characterized the structure of the N-NTD and its interaction with RNA using NMR spectroscopy. We observed a positively charged canyon on the surface of the N-NTD that might serve as a putative RNA binding site similarly to other coronaviruses. The subsequent NMR titrations using single-stranded and double-stranded RNA revealed a much more extensive U-shaped RNA-binding cleft lined with regularly distributed arginines and lysines. The NMR data supported by mutational analysis allowed us to construct hybrid atomic models of the N-NTD/RNA complex that provided detailed insight into RNA recognition.

## Author summary

The causative agent of the COVID-19 disease, the SARS-CoV-2 virus, has an unusually large genome that encodes for many proteins. Among them are four structural proteins (Spike, Membrane, Envelope and N proteins) important for RNA packing and virion assembly. Molecular understanding how new SARS-CoV-2 virions arise could direct new antiviral strategies urgently needed to combat the current pandemic. In our study, we describe how the N protein binds single- and double-stranded RNA, a key process for virion assembly. Our structural insights identified a large charged RNA binding groove on the surface of the N-terminal domain of the N protein that might play an important role in the higher-order supercoil structure formation in the context of the multiple copies of the dimeric full length nucleocapsid phosphoprotein.

## Introduction

The current COVID-19 pandemic is caused by the severe acute respiratory syndrome coronavirus 2 (SARS-CoV-2) of the *Coronaviridae* family [1]. The SARS-CoV-2 virus has already infected more than eight million people and caused the death of hundreds of thousands, overwhelming the global health care system capacity, disrupting our everyday lives and causing enormous economic damage that could develop into a deep economic crisis [2]. It also reminds us of the vulnerability of our civilization, characterized by high population density and intercontinental travel, to pathogens.

**Fig 1:**
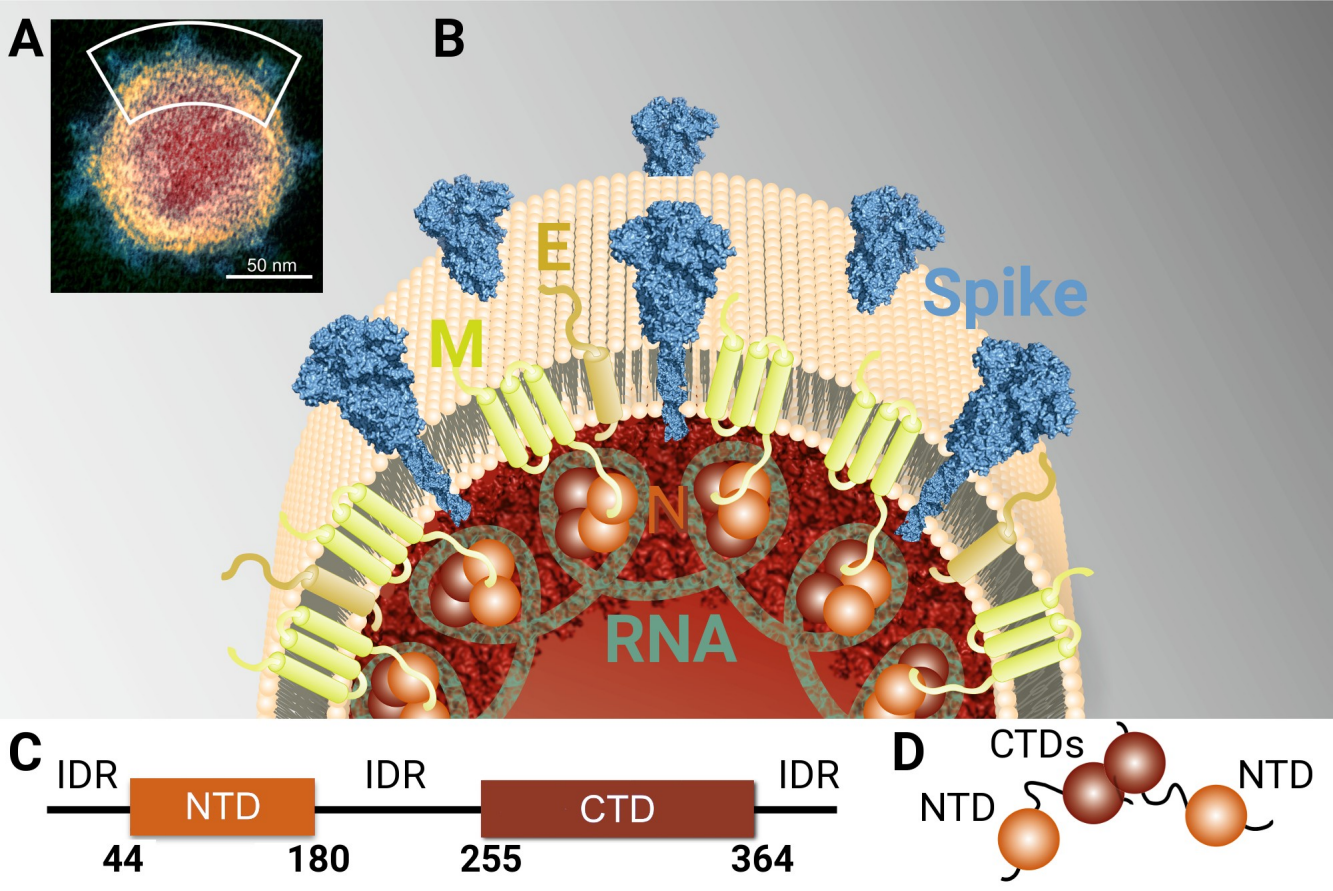
SARS-CoV-2 virion and model of structural proteins. (A) Transmission electron microscopic image of a single SARS-CoV-2 viral particle (image credit: NIH, NIAID-RML, https://www.niaid.nih.gov/news-events/novel-coronavirus-sarscov2-images). (B) Enlarged 2D model of the viral membrane showing the four structural proteins: Spike–Spike glycoprotein, M–Membrane protein, E–Envelope protein, and N–Nucleocapsid phosphoprotein along with viral membrane and the RNA genome. (C) Domain organization of the full length N-protein showing structural regions as boxes (NTD and CTD) and the intrinsically disordered regions (IDRs) as a line. (D) Schematic model of the full length N-protein dimer formed through the CTD domains (the N-NTD is shown in brown and the N-CTD in dark brown).

Like other coronaviruses, SARS-CoV-2 has an unusually large genome (29.8 kb) for a +RNA virus that encodes four structural proteins—the membrane (M), small envelope (E), spike (S) and nucleocapsid phosphoprotein (N)—and sixteen nonstructural proteins (nsp1-16) [3,4]. The nonstructural proteins bear all of the different types of enzymatic activity important for the viral proliferation, mostly associated with RNA replication. The SARS-CoV-2 genome also encodes an RNA-dependent RNA-polymerase complex (nsp7, nsp8 and nsp12), RNA capping machinery (nsp10, nsp13, nsp14 and 16) and additional enzymes such as proteases (the nsp3 PL^pro^ and the nsp5 3CL^pro^) which cleave viral polyproteins and/or impede innate immunity [4]. The four structural proteins together with the viral +RNA genome and the envelope constitute the virion. The membrane (M), small envelope (E), and spike (S) proteins are embedded within the lipid envelope [5]. The fourth structural protein, the nucleocapsid phosphoprotein (N), physically links the envelope to the +RNA genome, interacts with the endodomain of the viral membrane protein M [6] and plays a central role in the packaging signal RNA recognition and subsequent RNA encapsidation [7,8]. It consists of an N-terminal (NTD) and a C-terminal (CTD) domain (Fig 1) that are both capable of RNA binding. In addition, the CTD serves as a dimerization domain and the intrinsically disordered region (IDR) between the domains interact with the matrix protein forming the physical link between the +RNA genome and envelope. The SARS-CoV N protein has also been shown to modulate the host intracellular machinery and plays regulatory roles during the viral life cycle [9]. In light of the genomic similarities between SARS-CoV and SARS-CoV-2, it is reasonable to expect the SARS-CoV-2 N protein to function analogously.

All the SARS-CoV-2 enzymes are potential drug targets [10] and a detailed understanding of their functions is of the utmost importance. Recently, remdesivir, an RdRp inhibitor was approved by the FDA as an emergency treatment for severe COVID-19 cases. Remdesivir is a nucleotide analog, however, unlike most RNA viruses, SARS-CoV-2 encodes an exonuclease (a second enzymatic activity of the RNA capping factor nsp14) presumably capable of repairing mismatches in the newly synthesized double-stranded RNA. Additional antiviral compounds might be necessary to simultaneously target several viral proteins and create a trap that the virus cannot escape by mutation. In any case, drugs targeting proteins other than the RNA polymerase are urgently needed. In this study, we have analyzed in detail the structure of the N protein NTD (N-NTD) and its interaction with RNA using protein NMR. We combined the experimental data with computer simulations and devised a hybrid atomic model of the N-NTD and its complex with RNA that illustrates how the N protein recognizes single- and double-stranded RNA and reveals an RNA-binding groove that could serve as a pocket for inhibitor design.

## Results

### Structure of the SARS-CoV-2 Nucleocapsid Phosphoprotein N-terminal RNA binding domain (N-NTD)

**Fig 2:**
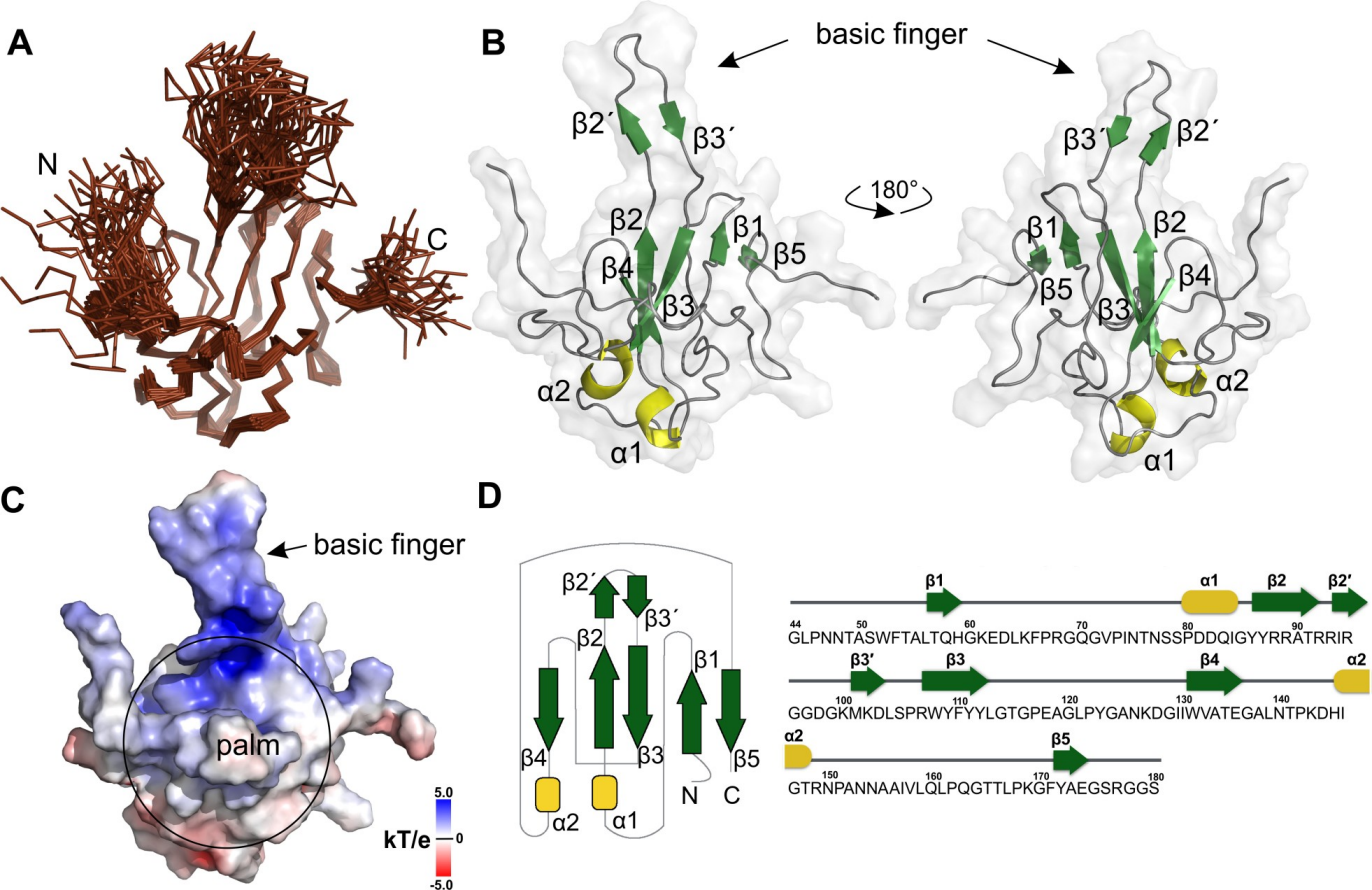
Solution structure of the SARS-CoV-2 N-NTD RNA binding domain. (A) Backbone representation of the 40 converged structures of N-NTD obtained by NMR spectroscopy. (B) Cartoon representation of the lowest energy structure (structural elements are highlighted in color: α1-α2 helices in yellow, β1-(β2’-β3’)-β5 in green, and loops in gray) show the overall U-shaped antiparallel β-sheet platform (the palm) and a protruding β-hairpin (the basic finger). (C) The N-NTD molecular surface electrostatic potentials revealed a basic patch extended between the finger and the palm, with a positively charged surface shown in blue and negatively charged surface in red. (D) Topology diagram of the N-NTD and protein sequence displaying the secondary structural elements.

We solved the NMR structure of the SARS-CoV-2 N-NTD domain. The structure revealed an overall right hand-like fold composed of a β-sheet core with an extended central loop. The core region adopts a five-stranded U-shaped right-handed antiparallel β-sheet platform with the topology β4-β2-β3-β1-β5 that is flanked by two short α-helices (α1 before β2 strand the and α2 after β5). A prominent feature is a large protruding loop between β2-β3 that forms a long basic β-hairpin (β2' and β3') (Fig 2). This long β-hairpin reminds a finger and is composed mostly of basic amino acid residues therefore we refer to it as a basic finger (Fig 2C). This basic finger is extending from the β-core structure that we further refer to as a palm. The analysis of electrostatic potential reveals a highly positively charged cleft between the basic finger and the palm creating the putative RNA binding site in the hinge/junction region between the palm and the basic finger in agreement with previous studies on other coronaviral N proteins [11–14]. Our NMR analysis is also consistent with the recent X-ray analysis (PDB IDs: 6M3M, 6VYO and 6WKP, S1 Fig) [15]. In addition, the NMR structure revealed that the basic finger is highly flexible (Figs 2A and S1) whereas in the crystal structures it is locked in one place by crystal lattice contacts.

### Confirming the putative RNA binding

**Fig 3:**
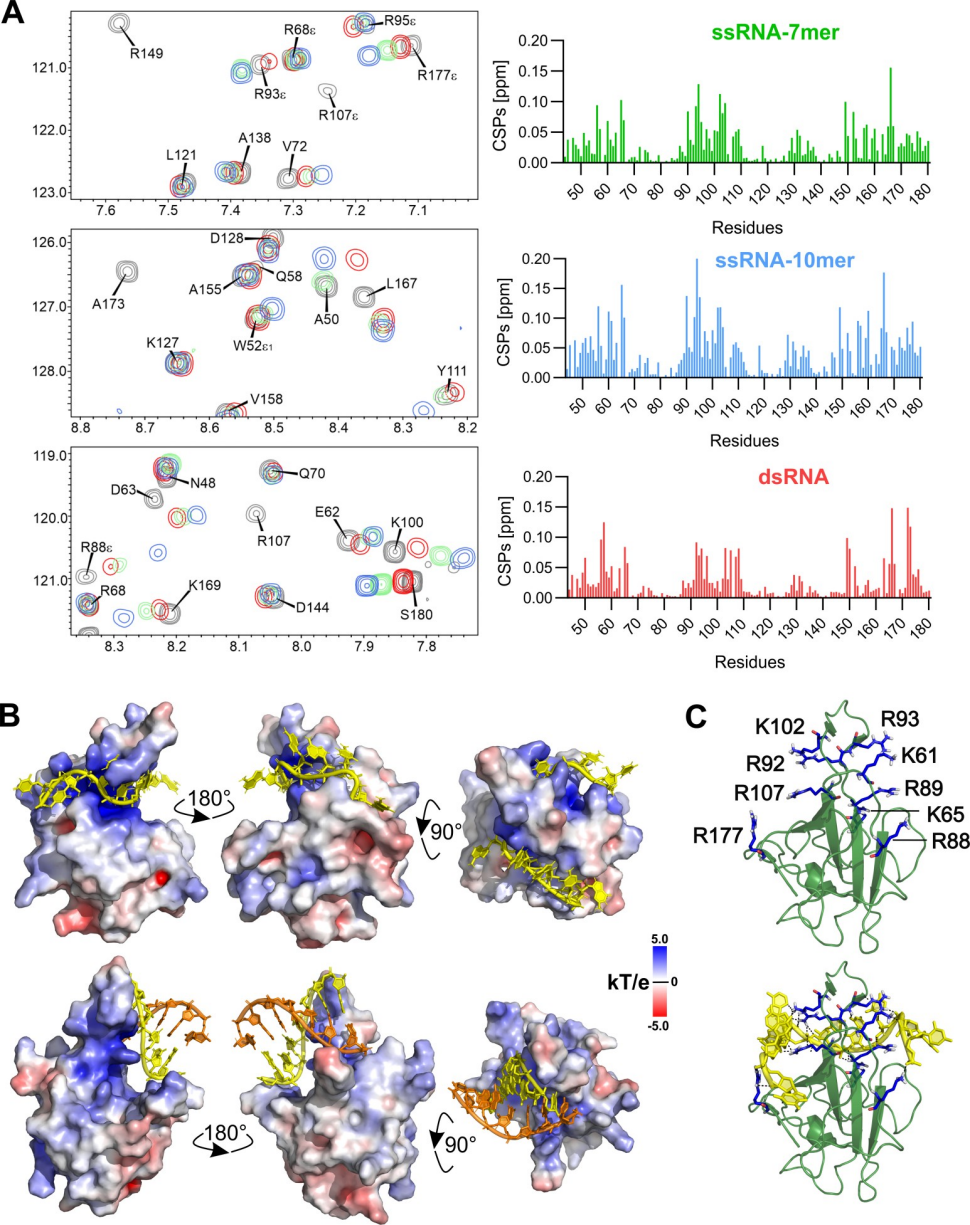
NMR-based mapping and a model of the SARS-CoV-2 N-NTD:RNA complex. (A) Representative regions from the 2D ^15^N/^1^H HSQC titration spectra illustrating the effects of addition of the RNA-7mer (green), 10mer (blue) and dsRNA (red) on the side-chain N-NTD amide signals (arginine side-chains are labeled along with NH^ε^). The 50 μM ^15^N-labeled N-NTD protein construct was titrated with an increasing concentration of RNAs. Corresponding chemical shift perturbations (CSP) of N-NTD residues upon binding ssRNA 7mer (5′-CUAAACG-3′) in green, 10mer (5′-UCUCUAAACG-3′) in blue from viral genomic 5′ UTR containing the conserved transcriptional regulatory sequence (TRS), and a random dsRNA (RNA-7mer duplex, 5′-CACUGAC-3′ and 5′-GUCAGUG-3′) in red. (B) N-NTD:RNA complex. The RNA-10mer and dsRNA are shown as a cartoon representation (yellow) over the electrostatic surface of N-NTD shown in three orientations. (C) Cartoon representation of N-NTD highlighting all the available arginine and lysine residues in the interaction interface, shown as blue sticks, and the lower panel displays the ssRNA-10mer docked model in same orientation.

Analysis of the surface electrostatic potential revealed a basic patch extending between the finger and the palm of the N-NTD suggesting a putative RNA binding site. We aimed to obtain experimental evidence of RNA binding to this site. We performed an NMR-based titration experiment using two single-stranded RNA (ssRNA) variants and a short double-stranded RNA (dsRNA). The 7mer (5'-CUAAACG-3') and the 10mer (5'-UCUCUAAACG-3') oligonucleotides were derived from the 5' untranslated region of the genomic SARS-CoV-2 RNA containing transcriptional regulatory sequence [16], while the dsRNA was a randomly chosen sequence stabilized by a G-C pair at both ends of the duplex (5'-CACUGAC-3' and 5'-GUCAGUG-3'). Basically, we were adding isotopically unlabeled RNA variants to the ^15^N/^13^C labeled protein and we followed changes in positions of the assigned signals in the NMR spectra (Fig 3) to reveal the molecular interface of the N-NTD:RNA complex. The high quality of the NMR data allowed for the unambiguous assignment of the arginine side-chain (NH^ε^) groups that were used together with the protein backbone amide signals for monitoring of RNA binding. Overlay of the 2D ^15^N/^1^H HSQC spectra of a free and RNA bound N-NTD revealed residues that were significantly perturbed by RNA interaction (S2 Fig). Both 7mer and 10mer ssRNA variants affected the same N-NTD residues. The higher chemical shift perturbations observed for the 10mer oligonucleotide reflect the increased affinity towards longer RNA. The significantly perturbed residues (L56, G60, K61, K65, F66, A90, R93, I94, R95, K102, D103, L104, T165, T166, G175 and R177) formed a U-shaped binding epitope on the N-NTD surface circumventing the base of the positively charged finger. The binding of dsRNA variant significantly affected residues A50, T57, H59, R92, I94, S105, R107, R149 and Y172 that are distributed in the basic finger or close to the junction between the basic finger and the palm as expected based on the analysis of the electrostatic potential.

### Structure of the N-NTD:RNA riboprotein complex

Next, we used the experimental data to build an atomic model of the protein:RNA complex. We used the HADDOCK protocol for the NMR-restraint driven docking simulations [17] of the relatively rigid dsRNA. However, this protocol did not yield satisfactorily converged structures for the complex formed by the highly flexible ssRNA oligonucleotide, as it could not be driven by ambiguous restraints to fully occupy the experimentally determined binding cleft. Therefore, we opted for an alternative real-time molecular dynamics simulation of the complex in YASARA [18] using NMR-derived distance restraints. For the HADDOCK simulation, we choose a short double helix based on the published crystal structure template of a short native RNA duplex [19] as a starting conformation of the dsRNA. Detailed analysis of the chemical shift perturbations (CSP) (Fig 3A) visualized on the solution structure obtained for N-NTD provided a set of ‘active’ solvent-accessible residues on N-NTD that were expanded for surrounding ‘passive’ residues. The selection criteria for active residues were that their CSP values were higher than 1.5x’s the standard deviation calculated for the entire set of CSPs and more than 20% solvent accessibility [20]. The restraints for the dsRNA molecule were kept ambiguous to avoid potential bias. The standard docking protocol yielded a set of water-refined conformations for the protein:dsRNA complex that were clustered into several distinct classes. As expected, the RNA duplex molecule was bound in the positively charged cleft in all the clusters (Fig 3B). The most populated cluster was also providing the least violations of experimental restraints and therefore it was selected as a representative conformation for the N-NTD:dsRNA complex. For the YASARA simulation of the N-NTD:ssRNA complex, we generated a network of distance restraints between the positively charged groups of the protein perturbed residues and negatively charged ssRNA backbone phosphate groups. Interestingly, the length of the U-shaped binding epitope outlined by NMR titrations is ~ 50Å, which corresponds to the length of the ssRNA-10mer molecule. In addition, detailed analysis of the perturbation data shown that the end of the binding cleft close to the N-terminus of N-NTD is formed by a positively charged lysine at position 65 that could form an electrostatic interaction with the 5’-end of ssRNA, while the opposite end of the cleft is mostly hydrophobic. The initial energy minimization was followed by 100 ns of molecular dynamics in the presence of intermolecular distance restrains that provided the final structure for the N-NTD:ssRNA complex (Fig 3B and 3C).

Our structural analysis revealed that both an RNA duplex and ssRNA bind in a similar manner to the positively charged canyon located between the basic finger and the palm of the N-NTD. The profound feature of the binding interface is its electrostatic potential. It is highly positive with several arginine residues (R92, R107 and R149) that directly bind the RNA. For the HCoV-OC43 N-NTD it was reported that R106, R107 and R117 (corresponding residues in SARS-CoV-2 are R92, R93 and K102) contribute to RNA binding while K110 (R95 in SARS CoV-2) does not [21] which is also predicted by our hybrid model. A study using the HCoV-NL63 N protein tested seven mutants and reported that all tested residues (Q59, R61, R63, K75, K77, R116, K121) contribute to RNA binding (these experimental data suggest somewhat different binding mode for HCoV-NL63 and HCoV-OC43 N-NTDs) [22]. The corresponding residues in SARS-CoV-2 are A90, R92, R95, R107, Y109, R149 and N154. For these our hybrid model predicts that A90 and R95 do not interact with RNA. We also did not observe any chemical shifts for R149 and N154 suggesting no interaction with RNA, however, here we cannot exclude that we would observe binding if we would use longer than 10mer RNA. Our model also explains the unspecific nature of N-NTD:RNA interaction. The N-NTD virtually only interacts with the RNA backbone while the bases are, in the case of ssRNA flipped away from the protein, or, in the case of dsRNA involved in base pairing but do not interact with the N-NTD domain (Fig 3B).

### RNA binding assay

**Fig 4:**
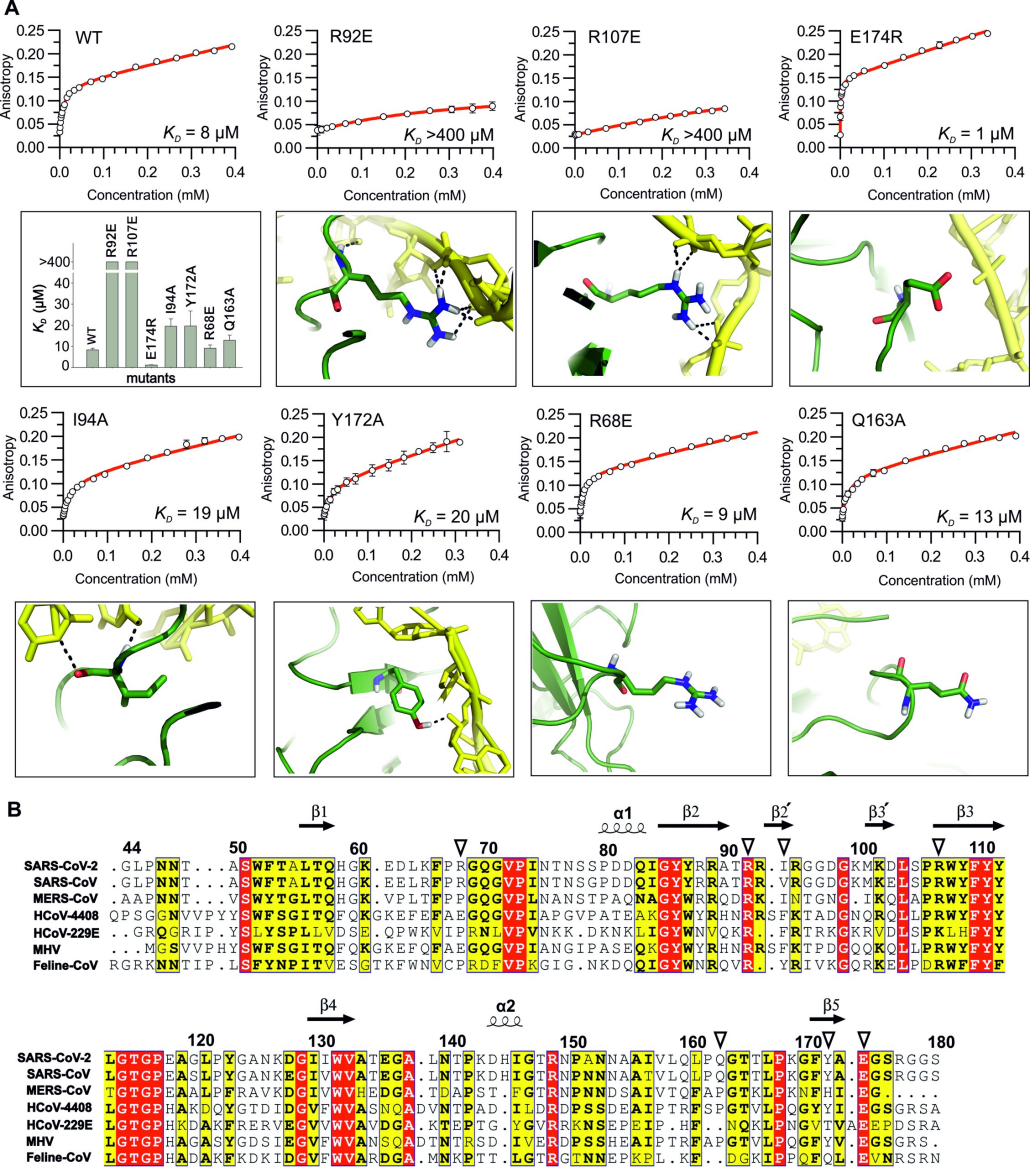
Mutational analysis of N-NTD:RNA interaction. (A) Binding curves N-NTD wild type and selected mutants (R92E, R107E, E174R, I94A, Y172A, R68E and Q163A) for RNA titrations obtained using the fluorescence anisotropy assay. Other panels display the zoom in view of the mutated residues showing hydrogen bonds between these residues and the RNA. A panel showing a plot comparing K_d_ values for the wild type and all mutants is also included. (B) Multiple sequence alignment of N-NTDs from SARS-CoV-2 and other selected coronaviruses, arrowheads highlight the residues selected for mutational analysis.

We used an RNA binding assays to gain a deeper insight into the interaction of the N-NTD with RNA. We titrated hexachlorofluorescein labeled ssRNA using the N-NTD domain and monitored the increase of fluorescence anisotropy. The RNA binding assay revealed that the wild type N-NTD binds the RNA with a K_d_ of 8.3 ± 0.8 μM (Fig 4A) under physiological salt concentration which is comparable to values previously obtained for nucleic acid binding (both RNA and DNA) of other coronaviral N-NTDs that were also reported to be in the low micromolar range [16,23,24]. The binding is strongest in a low salt (50 mM NaCl) buffer (K_d_ < 1 μM) and very weak (K_d_ > 400 μM) in high salt buffer (500 mM) illustrating the electrostatic nature of RNA binding by the SARS-CoV-2 N-NTD (S4 Fig).

### Mutational analysis of the N-NTD:RNA riboprotein complex

We used a structure-inspired mutational analysis to validate our model, first we selected two conserved arginine residues (R92 and R107, Fig 4B) that participate in the hydrogen bond network with RNA and we mutated them to charge swapping glutamate residues. Both mutants, R92E and R107E, showed essentially no binding to RNA (K_d_ > 400 μM) (Fig 4A, R92E and R107E panels). We noticed a negatively charged residue E174 that is located in close proximity to the RNA backbone and we also prepared a charge switch mutant, in this case E174R. This mutant showed almost an order of magnitude improvement in the RNA binding affinity (K_d_ = 1.2 ± 0.1 μM) presumably via electrostatic interaction as we introduced an additional positive charge near the RNA backbone (Fig 4A, E174R panel). To further validate our model, we also mutated two residues that according to our data moderately contribute to RNA binding to alanine residues (I94A and Y172A) which lead to a slight decrease of RNA binding affinity (K_d_ = 19.5 ± 3.5 and 19.6 ± 7.1 μM, respectively; Fig 4A, I94A and Y172A panels). In contrast to that, the mutation of residues that are according to our structural data not involved in the RNA binding (R68 and Q163) did not affect the RNA binding affinity (K_d_ = 9.1 ± 1.6 and 12.9 ± 2.4 μM, respectively; Fig 4A, R68E and Q163A panels).

## Discussion

Effective drugs are urgently needed to combat the COVID-19 disease. Most patients are not given any drug and the treatment relies on curing the symptoms. The most promising drug is remdesivir, a nucleotide analog that targets the viral RNA-dependent RNA-polymerase (RdRp). Viral polymerases are certainly good targets for antiviral compounds because these enzymes are absolutely vital for any +RNA virus. However, every viral enzyme is a potential target for antiviral compounds and an effective treatment may require several active compounds, each targeting a different protein at the same time. This approach, known as HAART (highly active antiretroviral therapy), has proven effective in the case of HIV, which is another virus with an RNA genome. In this study, we obtained a molecular snapshot of RNA recognition by the coronaviral N protein that revealed a deep charged canyon located in the interface of the basic finger and the palm that could be potentially used as a target for intervention by small molecules, albeit targeting structural proteins is always more difficult than targeting enzymes and the N-NTD is especially difficult target given its extensive surface area of the binding site.

Specifically, we obtained a hybrid atomic model of the N-NTD domain in complex with single-stranded and double-stranded RNA using computer simulations restrained by NMR data (chemical shift perturbations of backbone and side-chain amides upon RNA binding). The structure revealed a right-hand fold featuring a prominent basic finger protruding from the palm. Analysis of its electrostatic potential (Fig 2C) revealed highly positively charged canyon that is situated in the interface between the basic finger and the palm subdomain and constitutes a putative RNA binding site as was observed before for other related coronaviruses [11–14]. We performed an NMR titration experiment to obtain experimental proof of the RNA binding site. An overlay of the ^15^N/^13^C labeled protein NMR spectrum in the absence of ligand and in complex with RNA revealed amino acid residues with large chemical shifts upon the addition of RNA (Figs 3 and S2). Not surprisingly, all these residues are located in or in close proximity to the basic canyon, confirming the canyon as the RNA binding site. To illustrate how the coronaviral N-NTD recognizes RNA we built an atomic model of the N-NTD:RNA complex using the NMR titration data as an experimental restraint for computer simulations. The model reveals an unexpectedly large hotspot on the surface of the N-NTD spanning from the shallow pocket close to the N-terminus through the cleft between the finger and palm subdomains to the pocket next to the C-terminus. In order to satisfy all electrostatic contacts within the U-shaped binding interface, the ssRNA molecule forms essentially a half-turn, that might be the seeding step for the higher-order supercoil structure formation in the context of the multiple copies of the dimeric full length nucleocapsid phosphoprotein.

## Materials and methods

### Protein expression, and purification

DNA with coding sequence for SARS-CoV-2 N-NTD (residues 44–180) was obtained as an artificial gene (Thermo Scientific), cloned to pHIS-Parallel2 and expressed as a fusion protein with 6×His tag followed by cleavage site for TEV protease on its N-terminus. *Escherichia coli* BL21(DE3) expressing the protein minimal media containing ^15^NH_4_Cl and [U-^13^C]glucose (for NMR experiments) in 37°C and 220 rpm until OD_600_ reached 0.6. Then the expression was induced by adding 0.5 mM IPTG and the culture was further incubated shaking (220 rpm) for 16 h at 18°C. The cells were centrifuged (5000×g, 10 min, 4°C) and the pellet was lysed by sonication in lysis buffer (50 mM Tris pH 8.0, 500 mM NaCl, 20 mM imidazole, 10% glycerol, 3 mM β-mercaptoethanol) and the lysate was cleared by centrifugation (30000×g, 20 min, 4°C). His-tagged protein was purified from the supernatant by affinity chromatography on a Nickel-NTA (Machery-Nagel) according to the manufacturer's instructions, 6×His tag was cut off by TEV protease (1 μg of TEV added to 40 μg SARS-CoV-2 N-NTD and dialysed to lysis buffer at 4°C for 16 h). Protein sample was then passed through Nickel-NTA to remove the 6×His tag and uncut protein. The protein for NMR experiments was further purified by size-exclusion chromatography on a Superdex 75 HiLoad 26/60 column (GE Healthcare, USA) in buffer containing 20 mM Na_2_HPO_4_, 50 mM NaCl, 0.01% NaN_3_, pH 5.5. Purity of the protein was checked using SDS-PAGE. Protein was concentrated to 1.19 mM and used for NMR. For further NMR measuring of binding RNA, protein was diluted to 300 μM and flash frozen in liquid nitrogen and stored at -80°C.

To examine the RNA binding mode of the N-NTD we used ssRNA 7mer (5'-CUAAACG-3') and 10mer (5'-UCUCUAAACG-3') and dsRNA that was prepared by annealing of 7mer 5'-CACUGAC-3' and 5'-GUCAGUG-3' (Sigma) at the final concentration 200 μM of each oligonucleotide and water supplemented with 50 mM NaCl. The mixture was incubated at 60°C for 15 min and then cooled slowly at 26°C. For ssRNA titration, the ssRNA was added to 40 μM protein in molar ratios 1:0.25, 1:0.5, 1:0.75, 1:1, 1:2 and 1:4. For dsRNA titration, the annealed RNA was added to 50 μM protein in molar ratios 1:0.3125, 1:0.625, 1:1 and 1:2.

For fluorescence anisotropy assays, the protocol for expression and purification of SARS-CoV-2 N-NTD wild type and all mutants was the same as for the protein used for NMR, except the expression medium was LB medium and size-exclusion chromatography buffer was 10 mM Tris pH 8.0, 150 mM NaCl, 3 mM β-mercaptoethanol. Pure proteins were concentrated to ~2 mM.

### NMR spectroscopy

NMR spectra were acquired at 25°C on an 850 MHz Bruker Avance spectrometer, equipped with a triple-resonance (^15^N/^13^C/^1^H) cryoprobe. The sample volume was either 0.16 or 0.35 mL, in SEC buffer, 5% D_2_O/90-95% H_2_O. A series of double- and triple-resonance spectra [25,26] were recorded to obtain sequence-specific resonance assignment. We used the I-PINE assignment tool [27] implemented in NMRFAM-SPARKY [28] for initial automatic assignment. ^1^H-^1^H distance restraints were derived from 3D ^15^N/^1^H NOESY-HSQC and ^13^C/^1^H NOESY-HMQC, which were acquired using a NOE mixing time of 100 ms.

Structural calculation was carried out in CYANA [29] using NOESY data in combination with backbone torsion angle restraints, generated from assigned chemical shifts using the program TALOS+ [30]. First, the combined automated NOE assignment and structure determination protocol (CANDID) was used for automatic NOE cross-peak assignment. Subsequently, five cycles of simulated annealing combined with redundant dihedral angle restraints were used to calculate a set of converged structures with no significant restraint violations (distance and van der Waals violations < 0.5Å and dihedral angle constraint violations < 5°). The 40 structures with the least restraint violations were further refined in explicit solvent using the YASARA software with the YASARA forcefield [18] and subjected to further analysis using the Protein Structure Validation Software suite (www.nesg.org). The statistics for the resulting structure are summarized in Table 1. The structures, NMR restraints and resonance assignments were deposited in the Protein Data Bank (PDB, accession code: 6YI3) and BMRB (accession code: 34511).

**Table 1:** NMR Constraints and Statistics for the final set of structures.

Non-redundant distance and angle constrains	
Total number of NOE restraints	2405
Short-range NOEs	1281
Medium-range NOEs (1 < | i—j | < 5)	252
Long-range NOEs (| i—j | ≥ 5)	872
Torsion angles	176
Total number of restricting restraints	2581
Total restricting restraints per restrained residue	21.5
Residual constraint violations	
Distance violations per structure	
0.1–0.2 Å	2.8
0.2–0.5 Å	0.3
> 0.5 Å	0
r.m.s. of distance violation per constraint	0.01 Å
Maximum distance violation	0.29 Å
Dihedral angle viol. per structure	
1–10°	12.9
> 10°	2
r.m.s. of dihedral violations per constraint	0.49°
Maximum dihedral angle viol.	5.9°
Ramachandran plot summary	
Most favoured regions	87.0%
Additionally allowed regions	11.9%
Generously allowed regions	0.8%
Disallowed regions	0.3%
*r*.*m*.*s*.*d*. *to the mean structure*	*all/ordered*^*1*^
All backbone atoms	2.0/1.1 Å
All heavy atoms	2.1/1.5 Å
PDB entry	6YI3
BMRB accession code	34511

1 Residues with sum of phi and psi order parameters > 1.8

To follow changes in the chemical shifts of a protein upon RNA binding, we calculated chemical shift perturbations (CSPs). The CSP of each assigned resonance in the 2D ^15^N/^1^H HSQC spectra of the protein in the free state was calculated as the geometrical distance in ppm to the peak in the 2D ^15^N/^1^H HSQC spectra acquired under different conditions using the formula: $\Delta \delta =\sqrt{{\Delta \delta_{H}}^{2}+({\Delta \delta_{N}∙\alpha )}^{2}}$, where α is a weighing factor of 0.2 used to account for differences in the proton and nitrogen spectral widths [31].

### Molecular docking

The structure of the N-NTD in complex with the 7mer RNA duplex was calculated using HADDOCK [17]. The RNA homology model was prepared by mutating the native 7mer RNA duplex (PDB 4U37) [19] in Pymol (The PyMOL Molecular Graphics System, Version 2.0 Schrödinger, LLC.) that was subsequently subjected to an energy minimization in YASARA [18]. For the actual docking, we used a representative structure from the set of obtained structures and followed a standard protocol. As active were selected those N-NTD residues with CSP > 0.05 ppm and at least 20% solvent accessibility (A50, T57, H59, R92, I94, S105, R107, R149, A152 and Y172), while as passive were additionally selected adjacent solvent exposed residues (T49, T54, L55, R88, A90, K102, L104, Y109, Y111, P151, A155, A156, E174 and G175). On the RNA side, all 14 nucleobases were defined as active for the experimentally driven docking protocol. In addition, in all three regions within the N-NTD were defined as fully flexible segments for the advanced stages of the docking calculation (the N-terminal G1-T9, the central loop I54-M61 and the C-terminal S136-S140). The final set of 200 water-refined structures was clustered using a Fraction of Common Contacts approach [32] with a default cut-off 0.75 and a minimal cluster size = 4. The resulting structures were sorted into 7 clusters and the most populated cluster (n = 30) that also exhibited the lowest interaction energy was selected for detailed analysis. The structure of the N-NTD in complex with the ssRNA-10mer was calculated in YASARA [18]. The significantly perturbed backbone amide groups from residues (CSP > 0.06 ppm) N47, S51, F53, L56, G60, K61, K65, F66, A90, R93, I94, R95, G97, D98, K100, K102, D103, L104, G129, R149, A152, A156, I157, L159, Q160, T165, T166, L167, Y172, G175 and R177 outlined the U-shaped binding epitope for the ssRNA-10mer molecule. In addition, the signal from arginine side-chain NH^ε^ groups were significantly perturbed for residues 88, 89, 92 and 177 but remained at their original positions for 68, 93, 95. We combined this information in generating the inter-molecular distance restraints used in YASARA docking calculation that consisted of an initial energy minimization followed by a 100 ns of molecular dynamics using the default md_fast.mrc macro. The 3.9 Å upper distance limits were set between K65 NZ–U1 P, K61 NZ–U2 P, R88 NH1 –U5 P, R89 NH1 –U4 P, R92 NH1 –A6 P, K102 NZ–A7 P, R107 NH1 –A8 P, T166 CG2 –G10 C5, and R177 NH1 –C9 P and R177 NH2 –C10 P.

### RNA binding assays

The binding of wild type and all mutants of the SARS-CoV-2 N-NTD (residues 44–180) to RNA was measured using fluorescence anisotropy [33]. Briefly, fluorescently labeled RNA (UCUCUAAACG labeled with 5'-hexachlorofluorescein) was ordered from Sigma. The measurement was performed on an FluoroMax-4 spectrofluorometer (Horiba Scientific). The excitation wavelength was set to 538 nm and the emission wavelength to 553 nm. The concentration of RNA was 100 nM in the binding buffer (10 mM Tris pH 8.0, 150 mM NaCl, 3 mM β-mercaptoethanol) and the protein was titrated in the concentration range from 0 to 0.4 mM. The data were fitted in GraphPad Prism 8.4.2 using the One site—Total binding model.

### Sequence analysis

The protein sequences of N-NTD sequence from selected closely and distantly related to SARS-CoV-2 was retrieved from available PDB structures (SARS-CoV-2; 6YI3, SARS-CoV; 1SSK and MERS-CoV; 4UD1) and NCBI sequence (human enteric coronavirus HCoV-4408, AAQ67202; human coronavirus HCoV-229E, ARB07396; and murine hepatitis virus MHV, AAF05706; and Feline-CoV, ACS44223) databases, respectively. The multiple sequence alignment (MSA) of amino acid sequences was created using MAFFT v7 server (mafft.cbrc.jb/alignment/software). The final MSA of N-NTD figures with enhanced graphics showing color coded sequence similarities and secondary structural elements above the sequence derived from PDB ID:6YI3 was created using an online server Easy Sequencing in PostScript (ESpript) v3.0 (espript.ibcp.fr).

### Structural figures

All structural figures were prepared using PyMOL v2.3.5 (The PyMOL Molecular Graphics System, Schrödinger LLC, pymol.org).

## Accession codes

The NMR restraints, resonance assignments and the structure of the unliganded SARS-CoV-2 were deposited in the PDB under accession code 6YI3 and in the BMRB database under accession code 34511). The SARS-CoV-2 N-NTD in complex with the 7mer dsRNA under PDB accession code 7ACS and the complex with 10mer ssRNA under PDB accession code 7ACT.

## Supporting information

S1 Fig Structural and sequence alignment of our reported SARS-CoV-2 N-NTD NMR structure with other recently available N-NTD structures and with related coronaviruses namely SARS-CoV, MERS-CoV, Human Coronavirus (HCoV)-OC43, HCoV-NL63, Infectious Bronchitis Virus (IBV) and Mouse Hepatitis Virus (MHV). (A) Structural superimposition of SARS-CoV-2 N-NTD NMR structure PDB ID: 6YI3 (green), shown along with the backbone ribbon representation of 39 lowest energy NMR structure ensemble (light gray) aligned with 6M3M (purple), 6VYO (cyan) and 6WKP (yellow), illustrating the highly flexible basic finger subdomain and termini. (B) Superimposed Cα trace of currently available four SARS-CoV-2 N-NTD structures are represented as sausage model (ENDscript 2.0), where the radius is proportional to the deviation of r.m.s. between Cα pairs per residue between structures and white color shows the termini that is only present in the NMR structure. (C) Structural superimposition of SARS-CoV-2 N-NTD NMR structure (PDB ID: 6YI3) colored in green with SARS-CoV (1SSK—pink), MERS-CoV (4UD1—lilac), HCoV-OC43 (4J3K - orange), HCoV-NL63 (5N4K - purple), IBV (2GEC—blue), and MHV (3HD4—brown) with its respective electrostatic surfaces calculated for comparison. (D) Multiple sequence alignment of SARS-CoV-2 with other related coronaviral N-NTD with available structures and (E) Superimposed Cα trace of SARS-CoV-2 N-NTD NMR structure along with available coronaviral structures are represented as a sausage model, where the radius is proportional to the deviation of r.m.s. between Cα pairs per residue between structures and coloring based on sequence conservation (high-red to low-white).
(TIF)

S2 Fig NMR-HSQC spectral superimposition of free and RNA bound SARS-CoV-2 N-NTD. ^1^H-^15^N-HSQC spectral superimposition of free (dark blue) and RNA bound, which revealed specific chemical shift changes indicating the molecular interaction with RNA-10mer (light blue) and dsRNA (red), each labeled cross peak corresponds to the backbone or side-chain chemical shift of individual amino acid.
(TIF)

S3 Fig Chemical shift perturbations (CPS) upon RNA titration. CPS are shown as color coded intensity gradient for both complexes, In addition, residues that were used for docking of the 10mer ssRNA molecule are highlighted (A) using YASARA and 7mer dsRNA (B) using HADDOCK (for clarity, only residues used for construction of ambiguous restraints are shown).
(TIF)

S4 Fig Analysis of the effect of increasing ionic strength on RNA binding to SARS-CoV-2 N-NTD using fluorescence anisotropy assay. (TIF)
