# Supplementary figures and images for: Structural basis of RNA recognition by the SARS-CoV-2 nucleocapsid phosphoprotein
Source: PLoS Pathog. 2020 Dec 2;16(12):e1009100. doi: 10.1371/journal.ppat.1009100 (PMC7735635; doi:10.1371/journal.ppat.1009100)

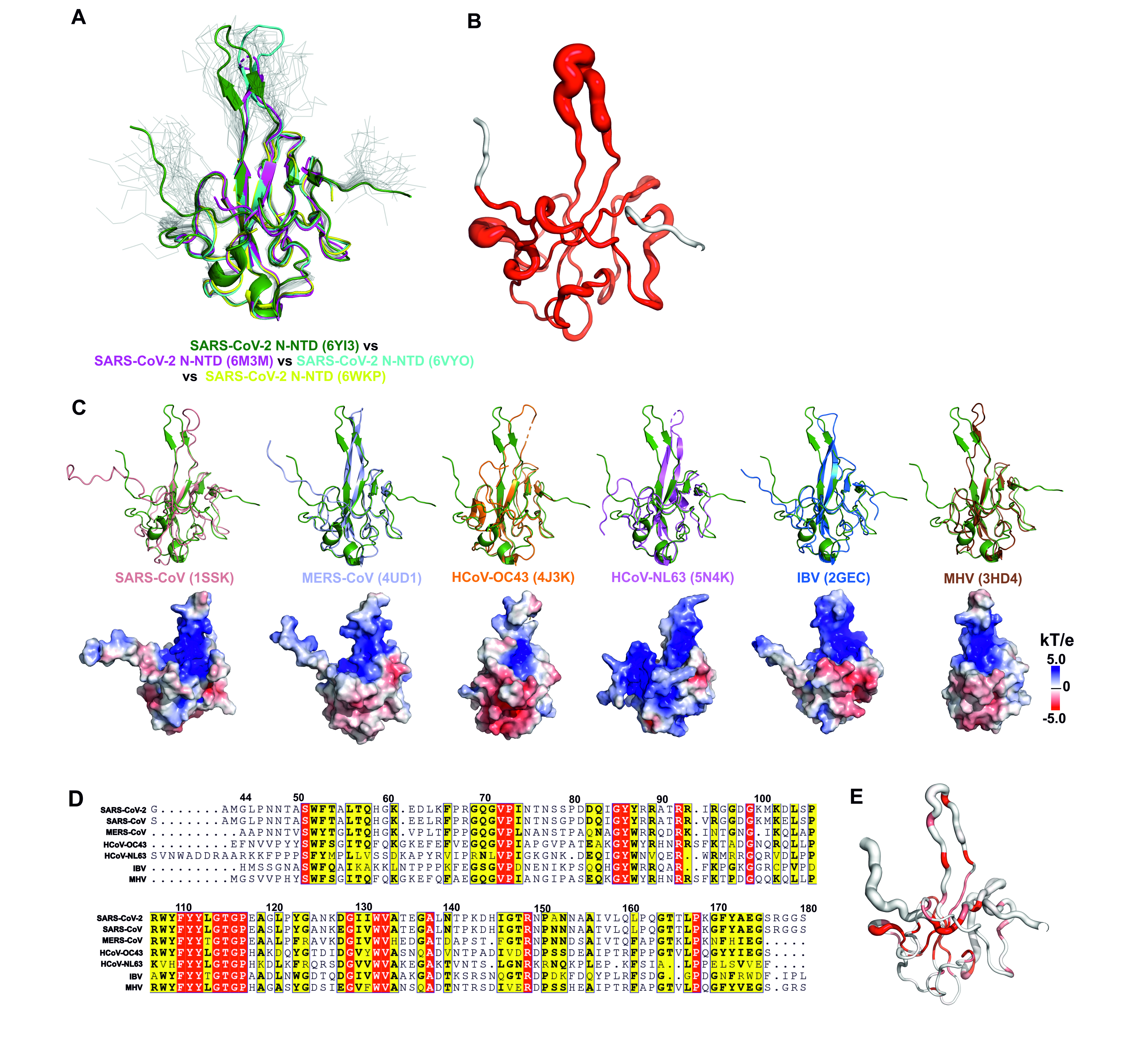

Supplement: S1 Fig — (A) Structural superimposition of SARS-CoV-2 N-NTD NMR structure PDB ID: 6YI3 (green), shown along with the backbone ribbon representation of 39 lowest energy NMR structure ensemble (light gray) aligned with 6M3M (purple), 6VYO (cyan) and 6WKP (yellow), illustrating the highly flexible basic finger subdomain and termini. (B) Superimposed Cα trace of currently available four SARS-CoV-2 N-NTD structures are represented as sausage model (ENDscript 2.0), where the radius is proportional to the deviation of r.m.s. between Cα pairs per residue between structures and white color shows the termini that is only present in the NMR structure. (C) Structural superimposition of SARS-CoV-2 N-NTD NMR structure (PDB ID: 6YI3) colored in green with SARS-CoV (1SSK—pink), MERS-CoV (4UD1—lilac), HCoV-OC43 (4J3K - orange), HCoV-NL63 (5N4K - purple), IBV (2GEC—blue), and MHV (3HD4—brown) with its respective electrostatic surfaces calculated for comparison. (D) Multiple sequence alignment of SARS-CoV-2 with other related coronaviral N-NTD with available structures and (E) Superimposed Cα trace of SARS-CoV-2 N-NTD NMR structure along with available coronaviral structures are represented as a sausage model, where the radius is proportional to the deviation of r.m.s. between Cα pairs per residue between structures and coloring based on sequence conservation (high-red to low-white). (TIF) [file ppat.1009100.s001.tif]

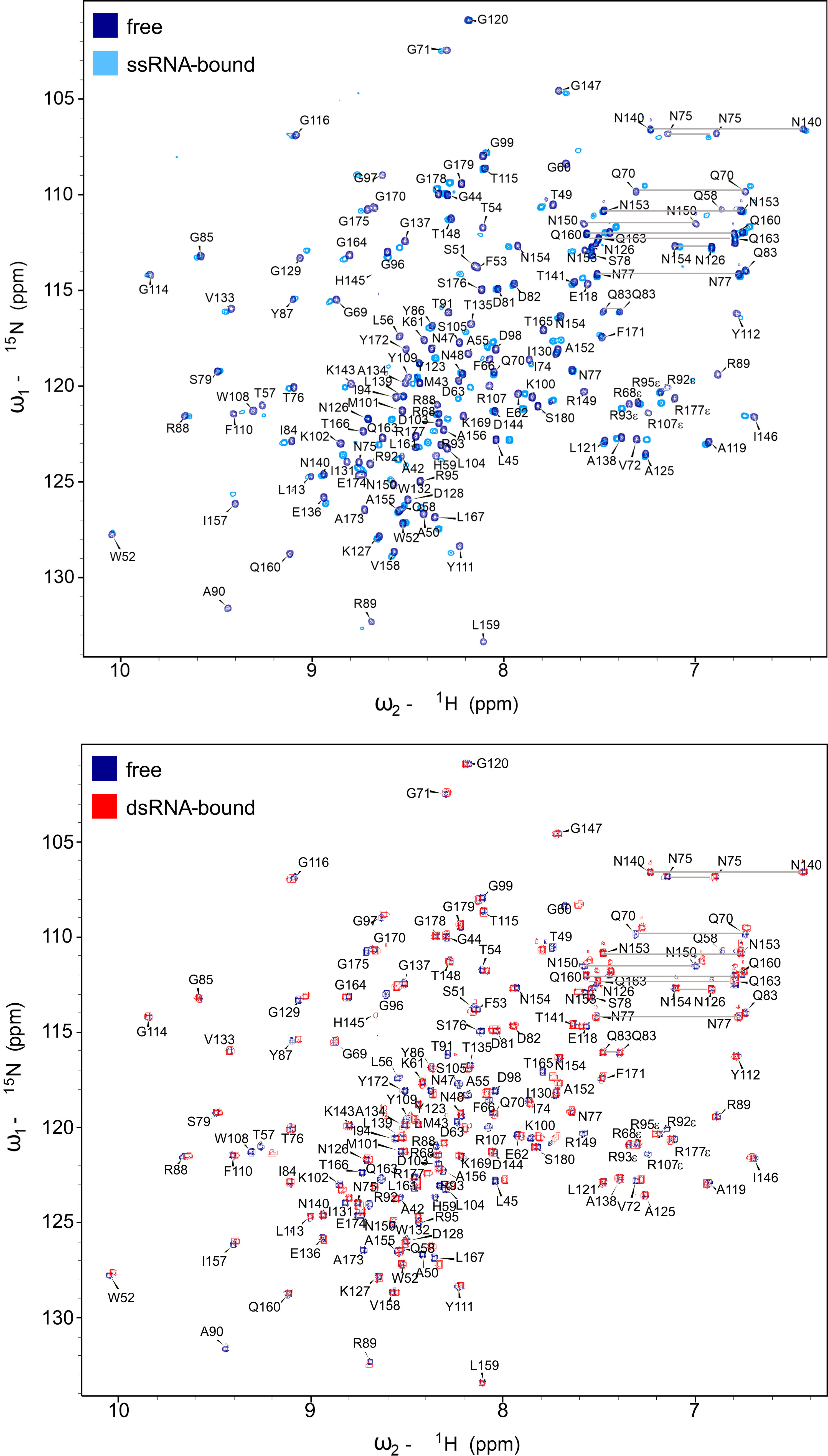

Supplement: S2 Fig — 1H-15N-HSQC spectral superimposition of free (dark blue) and RNA bound, which revealed specific chemical shift changes indicating the molecular interaction with RNA-10mer (light blue) and dsRNA (red), each labeled cross peak corresponds to the backbone or side-chain chemical shift of individual amino acid. (TIF) [file ppat.1009100.s002.tif]

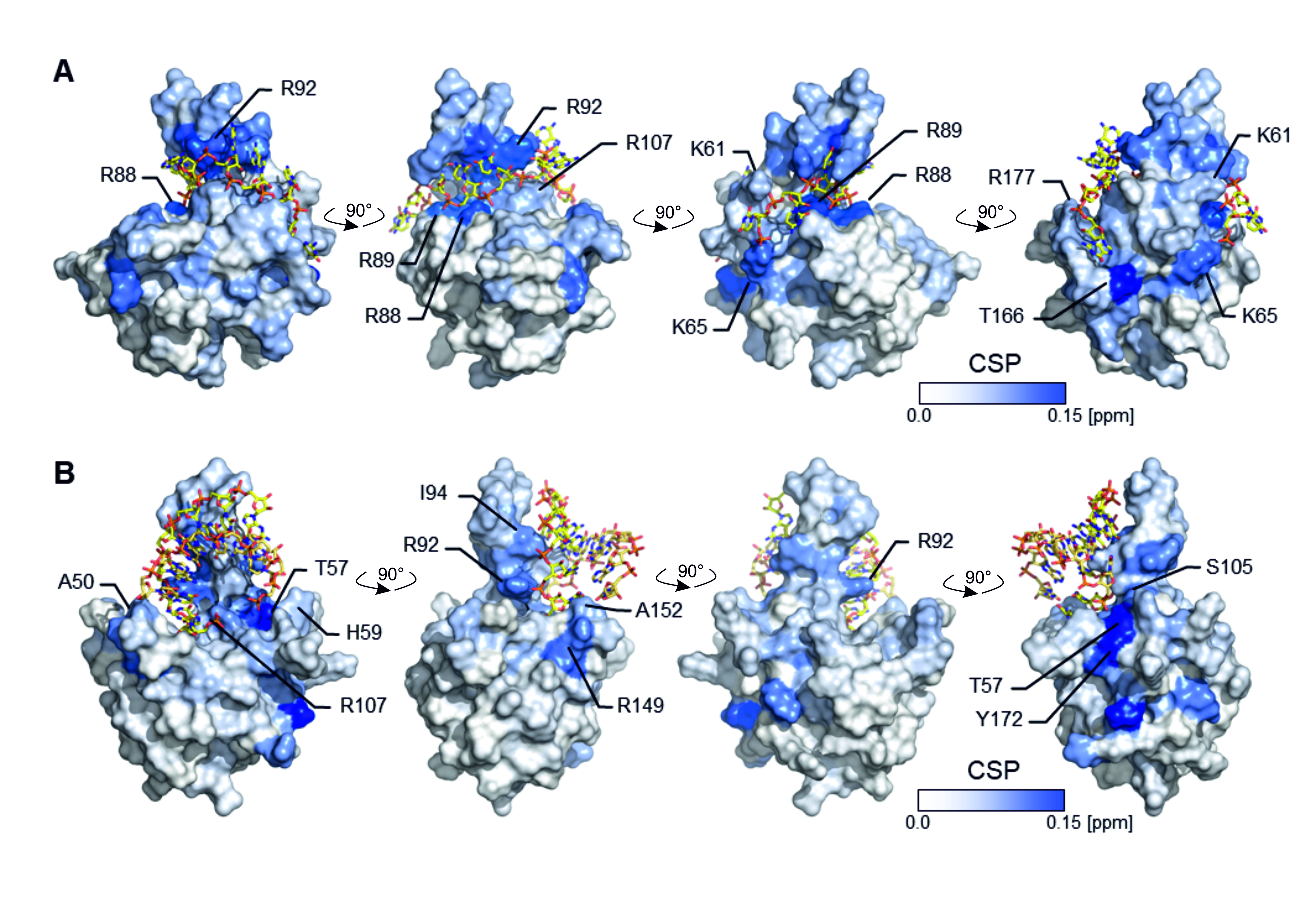

Supplement: S3 Fig — CPS are shown as color coded intensity gradient for both complexes, In addition, residues that were used for docking of the 10mer ssRNA molecule are highlighted (A) using YASARA and 7mer dsRNA (B) using HADDOCK (for clarity, only residues used for construction of ambiguous restraints are shown). (TIF) [file ppat.1009100.s003.tif]

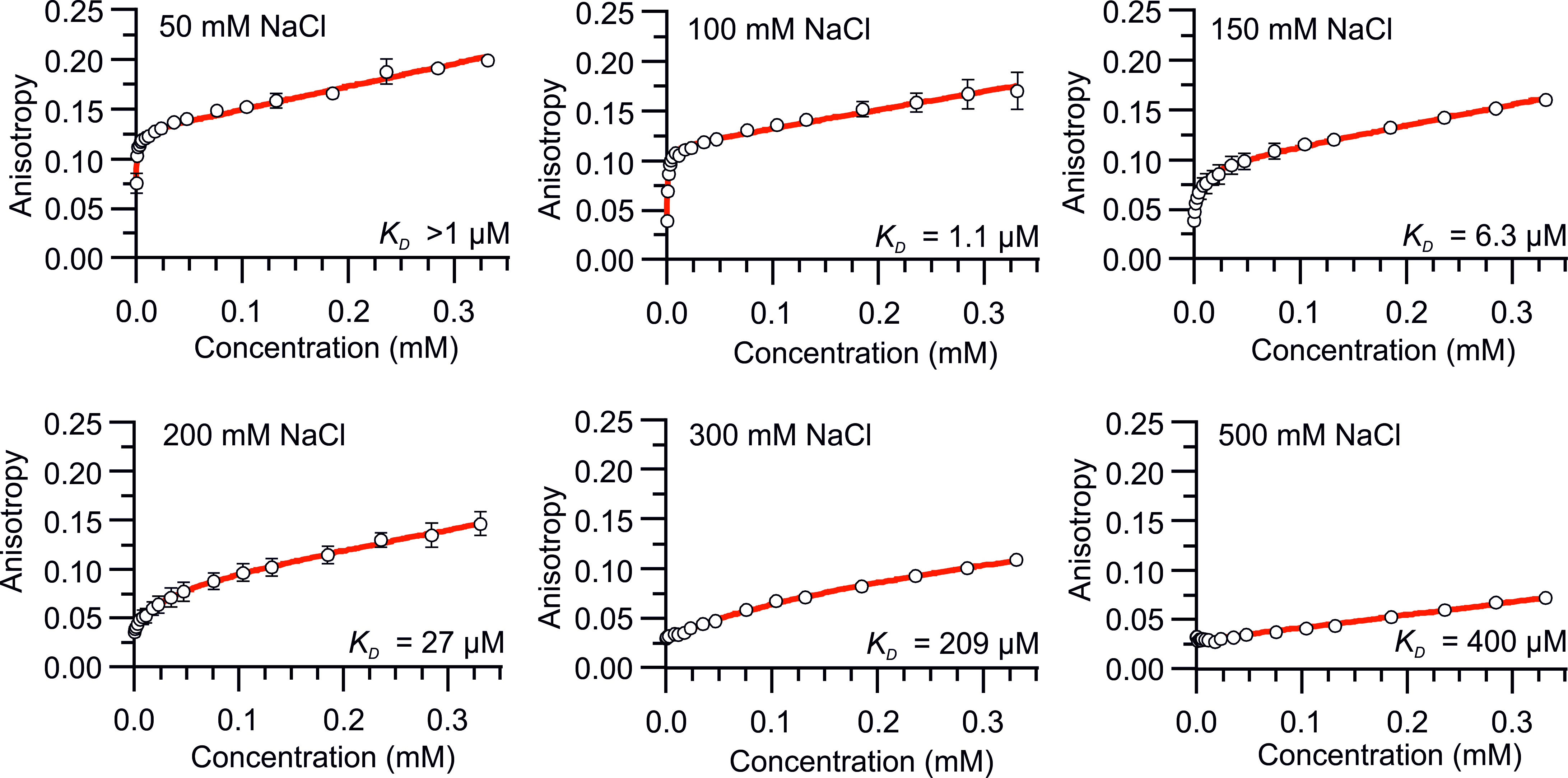

Supplement: S4 Fig — (TIF) [file ppat.1009100.s004.tif]
